# Gas analyzer's drift leads to systematic error in maximal oxygen uptake and maximal respiratory exchange ratio determination

**DOI:** 10.3389/fphys.2015.00308

**Published:** 2015-10-30

**Authors:** Ibai Garcia-Tabar, Jean P. Eclache, José F. Aramendi, Esteban M. Gorostiaga

**Affiliations:** ^1^Studies, Research and Sports Medicine Center, Government of NavarrePamplona, Spain; ^2^Laboratory of Performance, Sport-Occupational Activities-Biology-AssociationLyon-Chassieu, France

**Keywords:** exercise testing, maximal oxygen consumption, gas exchange, calibration, verification

## Abstract

The aim was to examine the drift in the measurements of fractional concentration of oxygen (FO_2_) and carbon dioxide (FCO_2_) of a Nafion-using metabolic cart during incremental maximal exercise in 18 young and 12 elderly males, and to propose a way in which the drift can be corrected. The drift was verified by comparing the pre-test calibration values with the immediate post-test verification values of the calibration gases. The system demonstrated an average downscale drift (*P* < 0.001) in FO_2_ and FCO_2_ of −0.18% and −0.05%, respectively. Compared with measured values, corrected average maximal oxygen uptakevalues were 5–6% lower (*P* < 0.001) whereas corrected maximal respiratory exchange ratio values were 8–9% higher (*P* < 0.001). The drift was not due to an electronic instability in the analyzers because it was reverted after 20 min of recovery from the end of the exercise. The drift may be related to an incomplete removal of water vapor from the expired gas during transit through the Nafion conducting tube. These data demonstrate the importance of checking FO_2_ and FCO_2_ values by regular pre-test calibrations and post-test verifications, and also the importance of correcting a possible shift immediately after exercise.

## Introduction

Maximal oxygen uptake ($\dot{\text{V}}{\text{o}}_{2\text{max}}$) is defined as the highest rate at which oxygen can be taken up and utilized by the body during exercise. In laboratory settings, $\dot{\text{V}}{\text{o}}_{2\text{max}}$ is commonly measured during incremental exercise to exhaustion, during which expired air is analyzed. The key variables needed to calculate $\dot{\text{V}}{\text{o}}_{2\text{max}}$ are the ventilator flow and the inspired and the expired fractional concentrations of oxygen (F_I_O_2_ and F_E_O_2_, respectively) and carbon dioxide (F_I_CO_2_ and F_E_CO_2_, respectively) (Hodges et al., 2005; Gore et al., 2013).

One of the main potential sources of error in the calculation of $\dot{\text{V}}{\text{o}}_{2\text{max}}$ using automated systems is related to the stability of F_E_O_2_ and F_E_CO_2_ measurements, because the electronic oxygen (O_2_) and carbon dioxide (CO_2_) analyzers are prone to drift over time (Winter, 2012; Gore et al., 2013). To our knowledge, there is surprisingly relatively little information available on the stability of O_2_ and CO_2_ analyzing systems over time during incremental exercise (Hodges et al., 2005; Salier Eriksson et al., 2012). In virtually all the publications that have measured $\dot{\text{V}}{\text{o}}_{2\text{max}}$, the authors have mentioned performing a pre-test calibration. As it has been pointed out in a recent Editorial (Winter, 2012), in the majority of these studies it is rare to see, however, equivalent post-test verifications. For instance, after reviewing more than 50 studies measuring $\dot{\text{V}}{\text{o}}_{2\text{max}}$ published between 1973 and 2012, we have found only 8 studies (~16%) in which the authors mentioned that the analyzers' drift at the completion of exercise was assessed (Wilmore et al., 1976; Armstrong and Costill, 1985; Prieur et al., 1998; McLaughlin et al., 2001; Rietjens et al., 2001; Day et al., 2003; Gore et al., 2003; Bowen et al., 2012). Only 4 of these 8 studies reported the average numerical drift values in O_2_% and CO_2_% (Wilmore et al., 1976; Armstrong and Costill, 1985; Prieur et al., 1998; Rietjens et al., 2001), which ranged from 0.02 to 0.22%. These reported drift values, according to the equations governing gas concentrations (Beaver et al., 1973; Wasserman et al., 1994a), would have caused an error in $\dot{\text{V}}{\text{o}}_{2\text{max}}$ up to 8–9% in standard laboratory conditions (~20°C of temperature, ~40% of relative humidity and ~720 mmHg of barometric pressure). Furthermore, none of these 4 studies gave any criterion for the maximum drift error that can be accepted. It is still unknown whether the drift magnitude is related to some physical or physiological exercise variables and how long any particular drift remains after the end of exercise. It is also unclear how the drift readings should be adjusted or corrected to overcome the inaccuracy due to the drift (Winter, 2012).

Clearly, it seems that the process of post-test verification tends to be overlooked and there is insufficient data available on how stable specific gas analysis systems are during exercise conditions (Atkinson et al., 2005; Salier Eriksson et al., 2012). This issue may be particularly relevant in several modern analyzers, in which the exhaled gas is not dried but is equilibrated with the laboratory environment by the use of a length of semi-permeable Nafion tubing (Medbø et al., 2002; Larsson et al., 2004). The purpose of the present study was, therefore, to examine the drift over time of a Nafion-using O_2_ and CO_2_ analyzing system during maximal incremental exercise in experienced athletes and elderly sedentary males. By including sedentary elderly and young athletic subjects, as well as short and long-duration exercise protocols, a large range of metabolic responses and exercise durations were examined and the influence of the drift on oxygen uptake ($\dot{\text{V}}{\text{o}}_{2}$), CO_2_ output ($\dot{\text{V}}{\text{co}}_{2}$) and respiratory exchange ratio (RER) assessment was determined. This study also proposed a way in which the error might be reduced.

## Materials and methods

### Subjects

Eighteen male amateur athletes (young group) and twelve older men (elderly group) volunteered to participate in the study. Athletes were recruited from various regional Sports Federations (Swimming, Athletics, Basketball, Basque-Ball, Paddle Tennis, Mountaineering and Climbing, Karate, Taekwondo, Judo, and Boxing). Athletes' mean (± SD) age, height, body mass,and percentage of body fat were 22 ± 6 years, 182 ± 7 cm, 79.3 ± 8.3 kg and 10.4 ± 3.1%, respectively. Participants in the elderly group were recruited from a Physical Activity Program for persons over 55. Mean (± SD) age, height, body mass, and percentage of body fat of the participants constituting the elderly group were 69 ± 6 years, 167 ± 7 cm, 85.9 ± 13.3 kg and 27.3 ± 4.3%, respectively. A detailed medical history was taken on the day of the study. No subject reported a history of abnormal dyspnea on exertion or of angina.

Written informed consent was obtained from all volunteers prior to their participation. The study was approved by the Institutional Review Committee of the Instituto Navarro del Deporte y Jueventud (Government of Navarre, Spain), according to the requirements of the Declaration of Helsinki.

### Exercise trials

Two different maximal incremental exercise protocols, with different exercise stage duration, were used for each population to examine whether the drift is influenced by the duration of the test. All testing sessions within each group were performed at the same time of the day in an air-controlled and well ventilated laboratory with a volume of 1121 m^3^. Young and elderly individuals reported to the laboratory at least 2 h after their last meal and having refrained from caffeine, alcohol, and strenuous or non-habitual exercise for 24 h before testing. Participants were habituated to the exercise testing equipment and procedures, as they were previously tested in the same laboratory using similar testing procedures.

#### Young exercise trials

Participants were habituated to the exercise testing equipment and procedures, as they were previously tested in the same laboratory using similar testing procedures. $\dot{\text{V}}{\text{o}}_{2\text{max}}$ was determined by a continuous maximal graded exercise test while sitting on a mechanically braked cycle-ergometer (Monark, Ergomedic 839-E, Varberg, Sweden). The exercise started at 20 W and the load was increased by 20 W every 2 min until volitional exhaustion. This exercise protocol was designed to reach volitional exhaustion within 23–33 min. It has been shown that relatively short (8–12 min) or long (~30 min) protocols do not affect attainment of $\dot{\text{V}}{\text{o}}_{2\text{max}}$ in highly motivated athletes (Gore et al., 2013). Participants maintained a constant cycling pedaling cadence of 60 rpm. Exhaustion was defined as the subject not being able to maintain the required pedaling cadence, despite vigorous verbal encouragement during the last min of exercise.

#### Elderly exercise trials

$\dot{\text{V}}{\text{o}}_{2\text{max}}$ was determined by a continuous incremental maximal exercise test on a treadmill ergometer (Kuntaväline, Hyper Treadmill 2040, Finland). The exercise test started at 5.5 km· h^−1^, after one min the speed was increased to 6.1 km· h^−1^ for another min, and thereafter grade was increased 1.1% every min until volitional exertion. Exhaustion was defined as the subjects not being able to maintain the required exercise intensity or they wished to stop.

At least two of the following criteria had to be met to determine $\dot{\text{V}}{\text{o}}_{2\text{max}}$ in both groups (American College of Sports Medicine, 2009): (1) no increase in $\dot{\text{V}}{\text{o}}_{2}$ despite increased workload, defined as a $\dot{\text{V}}{\text{o}}_{2}$ increment of less than 120 ml· min^−1^ per stage in the young group or a $\dot{\text{V}}{\text{o}}_{2}$ increment of less than 1.75 ml· kg^−1^· min^−1^ per stage in the elderly group. This criterion implies that any increment lower than 50% of the metabolic demand of these protocols' stages was accepted as a $\dot{\text{V}}{\text{o}}_{2}$ plateau (Taylor et al., 1955). (2) A maximal respiratory exchange ratio (RER_max_) greater than 1.10 (Robergs et al., 2010); (3) peak blood lactate concentration greater than 8 mmol·L^−1^, and (4) peak heart rate exceeding 90% of age predicted maximum (220-age). Heart rate (Polar Electro Oy, RS800CX, Kempele, Finland) was monitored throughout the exercise in both groups. Capillary blood samples from hyperemic earlobe were obtained at rest, on completion of the trial and at the 1st and 3rd min of recovery. After cleaning and puncturing, the single-use enzyme-coated electrode test strip was directly filled by a 5 μl whole-blood sample and blood lactate concentration was amperometrically determined (Arkray KDK Corporation, Lactate Pro LT-1710, Shiga, Japan).

### Collection of respiratory gases

Participants were fitted with an appropriately sized mouth and nasal breathing mask (Series 7930, Hans Rudolph, Kansas City, MO, USA) adjusted with a headgear (Vacu-Med, Ventura, CA, USA). Metabolic data was continuously collected using a Vista Mini-CPX (Vacu-Med, Silver Edition 17670, Ventura, CA, USA) computer-integrated metabolic system. The Vista Mini-CPX is a high precision mass flowmeter instrument composed of a turbine flow sensor and O_2_ and CO_2_ analyzers designed to measure the flow of the exhaled gases and the concentrations in the O_2_ and CO_2_ gases on-line. At the start of each test, room temperature (_R_T), barometric pressure (P_B_), and relative room humidity (_R_H) were measured (Precision Barometer, Lufft, Fellbach, Germany) and these data were entered manually into the computer. The environmental laboratory conditions were kept within the recommended values (18–23°C with a relative humidity lower than 70%) (Gore et al., 2013) by means of a heating system.

Minute expired ventilation (${\dot{\text{V}}}_{\text{E}}$) is calculated by a signal generated by the volume transducer of the turbine flow sensor. F_E_O_2_ is measured at _R_H through a disposable galvanic fuel cell (Teledyne Analytical Instruments, R-22MED Oxygen Sensor, Industry, CA, USA). F_E_CO_2_ is measured at _R_H through a nondispersive infrared system (Servomex, Ir1507 CO_2_ infrared transducer, Crowborough, UK). According to the manufacturer, the CO_2_ and O_2_ analyzers have zero drift (< 1.5 Torr in 1 h for the CO_2_ analyzer and 0.3% a week at constant temperature for the O_2_ analyzer) and their response times are 90 to 130 ms (CO_2_ analyzer) and 5 s (O_2_ analyzer). This time delay is automatically assessed and the length of the airline is taken into account according to the manufacturer's specifications. From these measurements the metabolic cart's computer calculates the mass flow of $\dot{\text{V}}{\text{o}}_{2}$ (in liters per minute), $\dot{\text{V}}{\text{co}}_{2}$ (in liters per minute), and the ratio of $\dot{\text{V}}{\text{o}}_{2}$ to $\dot{\text{V}}{\text{co}}_{2}$ (RER) with an accuracy (according to the manufacturer) of ±1% in measures of F_E_O_2_ and F_E_CO_2_, of ±2% in measures of ${\dot{\text{V}}}_{\text{E}}$, and of ±3% in measures of $\dot{\text{V}}{\text{o}}_{2}$ and $\dot{\text{V}}{\text{co}}_{2}$.

This metabolic system uses a proportional sampling approach in the process of mixing the exhaled gases. Thus, the flow rate of this sampling is closely related to the flow of exhalation at ~0.5% of its rate, and directs the exhaled gases in three steps into the O_2_ and CO_2_ gas analyzers connected in parallel: (1) through a capillary tube, into a miniature mixing chamber, (2) through a built-in Nafion gas dryer humidifier conducting 180 tube (29 cm long × 1 mm inner diameter), and (3) through a capillary tube system with the same configuration of sampling tube length, diameter and pump flow rate for both analyzers. The Nafion tube is a semi-permeable membrane to water vapor made of copolymer of tetrafluoroethylene (Teflon®) and perfluoro-3,6-dioxa-4-methyl-7-octene-sulfonic acid, highly selective in the removal of water from the vapor phase. The Nafion tube allows water vapor to pass in and out of the tube by absorption and conveys the exhaled gases to the gas analyzers once an equilibrium is reached with the ambient humidity (Macfarlane, 2001). According to the Nafion manufacturer, during exercise the water vapor tension of the aspirated gas sample (relative humidity ~100%) (Bageant, 1976; Macfarlane, 2001; Atkinson et al., 2005) is reduced in milliseconds to the level of _R_H of the laboratory environment (~27%, Table 1) by moving the water through the Nafion membrane wall and evaporating it very quickly into the surrounding air. Conversely, the typically dry calibration gas is humidified by the Nafion tubing to the level of _R_H. This system provides a constant value of water vapor tension of the exhaled and calibration gases just prior to the entry of the samples into the gas analyzers. The Nafion tube was replaced at least every 3 years according to the manufacturer. All tests were carried out within the 18 months following the last Nafion tube replacement.

**Table 1:** **Room environmental conditions (mean ± SD) during the exercise and non-exercise trials**.

	**Young exercise trials (*N* = 18)**	**Young non-exercise trials (*N* = 18)**	**Elderly exercise trials (*N* = 12)**	**Elderly non-exercise trials (*N* = 12)**
Temperature (°C)	21.0 ± 1.2	20.4 ± 0.5	20.4 ± 0.5	20.1 ± 0.3
Humidity (%)	27 ± 6	28 ± 4	28 ± 4	24 ± 1
Pressure (mmHG)	726 ± 5^*^	716 ± 4	716 ± 4	715 ± 4

* *Significantly different from the simulated trials; P < 0.01*.

The metabolic measurement software supplied with the analyzer (Vacu-Med, TurboFit 5, Ventura, CA, USA) was set to report mean metabolic data over a 30 s time period and to adjust the volume of the expired air to standard conditions (STPD) for temperature (0°C), pressure (760 mmHg), and dry (absence of water vapor). $\dot{\text{V}}{\text{o}}_{2\text{max}}$ was defined as the highest 30-s $\dot{\text{V}}{\text{o}}_{2}$ value averaged over two consecutive readings, and its time-corresponding values of $\dot{\text{V}}{\text{co}}_{2}$, ${\dot{\text{V}}}_{\text{E}}$ and RER were considered as $\dot{\text{V}}{\text{co}}_{2\text{max}}$, ${\dot{\text{V}}}_{{\text{E}}_{\text{max}}}$, and RER_max_, respectively.

### Pre-test calibration and post-test verification processes

The instrument was warmed up for at least 2 h prior to every exercise test to minimize any possible electrical drift. Calibration of the O_2_ and CO_2_ analyzers was performed immediately prior to every test using two-point calibration with two precision-analyzed gas mixtures. One calibration point was room air (O_2_: 20.93%; CO_2_: 0.00%) Non-hygroscopic soda lime CO_2_ absorbent (Vacu-Med, Ventura, CA, USA) was used for maximum precision of ambient CO_2_ measurement. Thus, fractional concentrations of room air were assumed to be 20.93% O_2_ and 0.00% CO_2_. The second point was a high-precision certified calibration tank gas containing 15.05% O_2_, 5.99% CO_2_ and balanced nitrogen. This high-precision gas was determined gravimetrically, was obtained from a reliable gas supplier (Praxair, Madrid, Spain) and had a claimed accuracy of ±0.02%. Turbine flow calibration was determined using a high-precision 3-L calibration syringe (Vacu-Med, Calibringe 1092, Ventura, CA, USA), in a five-pump series. A series of complete pumps of the syringe and of gas calibrations were repeated until the difference between the current and the previous calibration was less than 0.05 L for volume and less than 0.02% for O_2_ and CO_2_. When the calibration process was finished, the gas sample line was connected to the subject's mask.

Within 15 s of the completion of each exercise trial the sample line was removed from the connection to the face mask/turbine and the after trial verification of FO_2_, FCO_2_ and turbine flow measurements was performed. Both calibration gases (room air and tank gas) were run through the metabolic system to check for the drift of the analyzer over the course of the measurement period. Verification readings of the calibration gases and the flow sensor were noted down and compared with the calibration references.

### Correction of metabolic data

Post-test verifications readings were used to correct the metabolic data measured by the Vista Mini-CPX. Corrected ${\dot{\text{V}}}_{\text{E}}$ (${\dot{\text{V}}}_{{\text{E}}_{\text{C}}}$) in STPD condition was calculated as follows:

$$\begin{array}{l} {\dot{\text{V}}}_{{\text{E}}_{\text{C}}}=3\cdot {\dot{\text{V}}}_{{\text{E}}_{\text{me}}}\cdot {\left[\text{Cal}+\left(\text{Ver}-\text{Cal}\right)\right]}^{-1}\\ {\dot{\text{V}}}_{{\text{E}}_{\text{C}}}=3\cdot {\dot{\text{V}}}_{{\text{E}}_{\text{me}}}\cdot {\left[\text{Ver}\right]}^{-1} \end{array}$$

where “3” was the volume (L) of the syringe used to calibrate the flow sensor, “${\dot{\text{V}}}_{{\text{E}}_{\text{me}}}$” was the minute ventilation (L· min^−1^) in STPD condition measured by the metabolic cart, “Cal” was the calibration readout (L) recorded before the exercise and “Ver” was the verification readout (L) recorded after the exercise.

**Figure 1:**
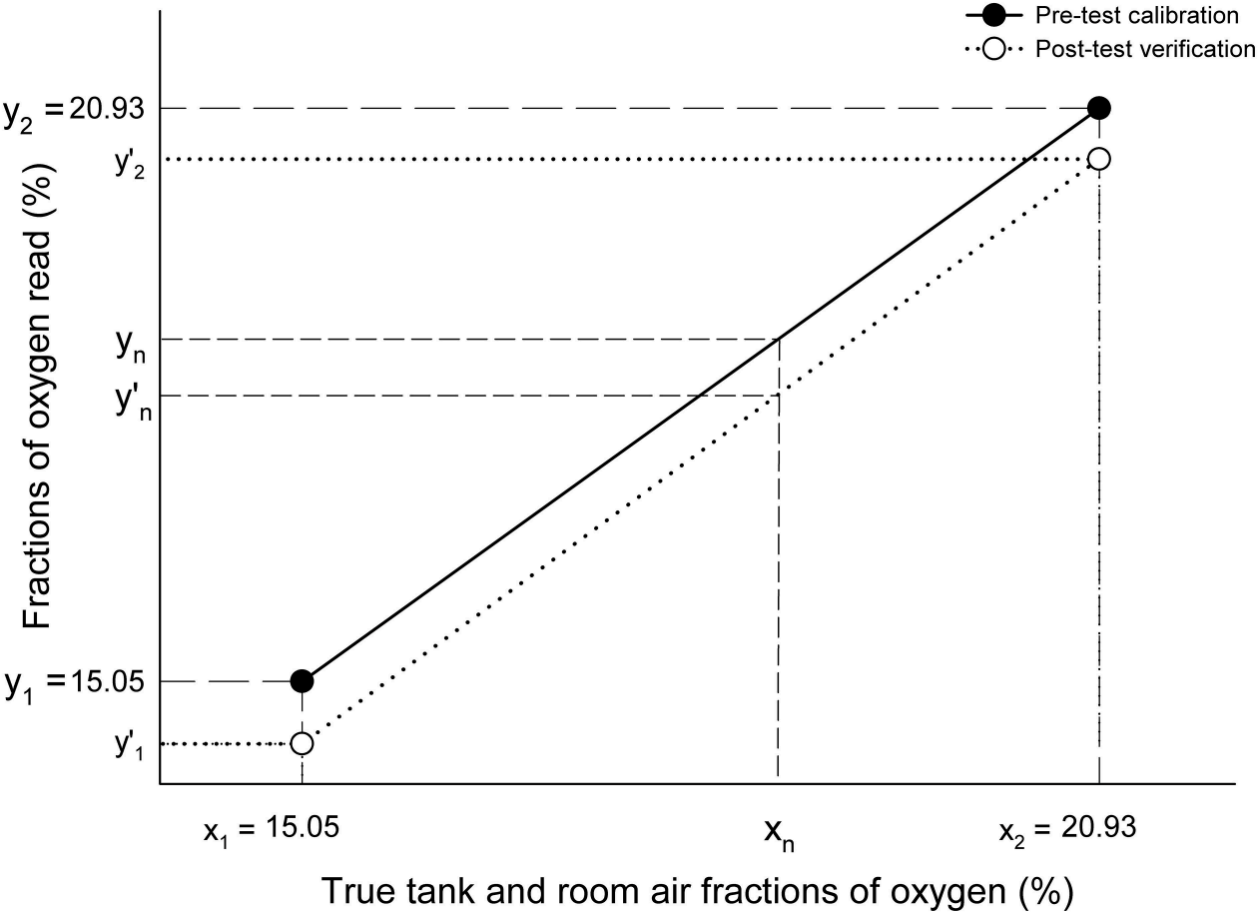
**Correction of fractional concentrations of oxygen**. x_1_ and x_2_, true tank (*x*_1_ = 15.05%) and room air (*x*_2_ = 20.93%) fractions of oxygen; y_1_ and y_2_, fractions of tank (*y*_1_ = 15.05%) and room oxygen (*y*_2_ = 20.93%) read by the analyzer during the pre-test calibration process when the true tank (x_1_) and room air (x_2_) gases were aspirated by the analyzers; y′_1_ and y′_2_, fractions of tank (y′_1_), and room oxygen (y′_2_) read by the oxygen analyzer during the post-test verification process when true tank (x_1_) and room air (x_2_) gases were aspirated by the analyzers.

Correction of FO_2_ is illustrated in Figure 1. During the pre-test calibration process we adjusted the gain settings of the span potentiometers to the corresponding voltage outputs, so that readings of O_2_% (tank gas: y_1_ = 15.05%; room air: y_2_ = 20.93%) equaled real O_2_% (x_1_ = y_1_; x_2_ = y_2_). The equation of the pre-test calibration regression line is therefore:

$$\begin{array}{l} \text{Y}=\text{X} \end{array}$$

During the post-exercise verification process we used the same pre-test calibration gases (x_1_ = 15.05% O_2_; x_2_ = 20.93% O_2_), but the %O_2_ values read (y′_1_ and y′_2_) were different from the O_2_% read during the pre-test calibration process. In this case, the equation of the post-test verification regression line is:

$$\begin{array}{l} Y=A^{\prime }\cdot X+B^{\prime } \end{array}$$

Being:

$$\begin{array}{l} A^{\prime }=\left(y_{2}^{\prime }-y_{1}^{\prime }\right)∕\left(x_{2}-x_{1}\right)\\ B^{\prime }=y_{2}^{\prime }-\left(A^{\prime }\cdot {\text{x}}_{2}\right) \end{array}$$

For a given value of (y′_n_) measured at ${\dot{\text{Vo}}}_{2\text{max}}$ during exercise, we can calculate the corresponding value of x (x_n_) from the equation of the post-test verification line (Y = A′X + B′) as follows:

$$\begin{array}{l} y_{\text{n}}^{\prime }=A^{\prime }\cdot {\text{x}}_{\text{n}}+B^{\prime }\\ {\text{x}}_{\text{n}}=\left(y_{\text{n}}^{\prime }-B^{\prime }\right)∕A^{\prime } \end{array}$$

Therefore, the corrected F_E_O_2_ value at $\dot{\text{V}}{\text{o}}_{2\text{max}}$ (y′_n_) in the pre-test calibration line (Y = X) is:

$$\begin{array}{l} y_{\text{n}}^{\prime }={\text{x}}_{\text{n}} \end{array}$$

F_E_CO_2_ was corrected using this same procedure. Once the corrected F_E_O_2_, F_E_CO_2_, and ${\dot{\text{V}}}_{{\text{E}}_{\text{C}}}$ were obtained, formulas provided by the manufacturer [see Beaver et al. (1973) or Wasserman et al. (1994a) for further detail] were employed to correct $\dot{\text{V}}{\text{o}}_{2}$ and $\dot{\text{V}}{\text{co}}_{2}$ as follows:

$$\begin{array}{l} \dot{\text{V}}{\text{co}}_{2}=\left({\text{F}}_{\text{E}}{\text{CO}}_{2}-{\text{FiCO}}_{2}\right)\cdot {\dot{\text{V}}}_{{\text{E}}_{\text{C}}}\cdot \text{HF}\\ \dot{\text{V}}{\text{o}}_{2}=\left[{\text{FiO}}_{2}\cdot {\text{FeN}}_{2}\cdot {\left({\text{FiN}}_{2}\right)}^{-1}-{\text{F}}_{\text{E}}{\text{O}}_{2}\right]\cdot {\dot{\text{V}}}_{{\text{E}}_{\text{C}}}\cdot \text{HF} \end{array}$$

where FiCO_2_, FiO_2_, and FiN_2_ are fractions of inspired carbon dioxide, oxygen and nitrogen respectively, F_E_CO_2_, F_E_O_2_, and FeN_2_ are fractions of corrected expired carbon dioxide, oxygen and nitrogen respectively, ${\dot{\text{V}}}_{{\text{E}}_{\text{C}}}$ is the corrected minute ventilation (L·min^−1^) in STPD condition, and HF is the humidity factor defined as:

$$\begin{array}{l} \text{HF}={\text{P}}_{\text{B}}-{\text{PH}}_{2}\text{O}\left(\text{at}{\;}_{\text{R}}\text{T}{,}_{\text{R}}\text{H}\right)\cdot {\left({\text{P}}_{\text{B}}\right)}^{-1} \end{array}$$

where P_B_ is the barometric pressure (mmHg) and PH_2_O is the pressure of water (mmHg) at room temperature (_R_T) and humidity (_R_H). Standard tables provided by the manufacturer, also presented by Wasserman et al. (1994b), were used to determine PH_2_O.

The metabolic system calculates FiN_2_ and FeN_2_ using the next two formulas:

$$\begin{array}{l} {\text{FiN}}_{2}=0.79\cdot \text{HF}\\ {\text{FeN}}_{2}=\text{HF}-{\text{F}}_{\text{E}}{\text{O}}_{2}-{\text{F}}_{\text{E}}{\text{CO}}_{2} \end{array}$$

where it is assumed that FiN_2_ is constant and FeN_2_ is the remaining fractional gas of HF, F_E_O_2_ and F_E_CO_2_.

All corrections were performed off-line using specific routines developed in a commercial software package (The MathWorks Inc., MATLAB R2008a, Natick, MA, USA).

### Non-exercise trials

To check the stability of the analyzers, each exercise test was pair-matched on duration, time of the day and number of pre-test calibrations and post-test verifications assessed, with a non-exercise trial, accounting for a total of 30 non-exercise trials (one per subject). These non-exercise trials consisted of performing the identical calibration and verification processes of the gas analyzers over the same time interval to that used during each exercise trial. Between the calibrations and verifications, the metabolic system worked throughout but no subject was connected to the metabolic cart. No flow or volume measures were recorded.

### Recovery trials

The pattern of change in FO_2_ and FCO_2_ during the first 30 min of recovery after the completion of the exercise trials, and after disconnecting the gas sample line from the mask, was investigated immediately after 9 exercise trials. These recovery trials consisted of performing the post-test verifications of the gas analyzers within 15 s of the completion of each exercise trial, but also at 3, 5, 10, 15, 20, and 30 min of recovery from each exercise trial.

### Statistics

Standard statistical methods were used for the calculation of means, standard deviations (SD), standard errors of the estimates (SEE), and confidence intervals (CI). Data were analyzed using parametric statistics following confirmation of normality, homoscedasticity, and when appropriate sphericity. Gas measure readings after the trials (verification readings) were compared with the concentrations of the standard calibration gases (calibration readings) using two-tailed one-sample Student's *t*-tests. Two-tailed Student's paired *t*-tests were used to analyze differences between verification readings of the exercise trials and their paired non-exercise trials, as well as between the non-corrected (measured) and corrected values of the respiratory parameters. Respiratory values of the elderly and young groups were compared by two-tailed independent samples *t*-tests, with Levene's tests used to assess equality of variances. Relationships between variables of interest were assessed by linear regression analyses. Pearson product-moment correlation coefficients (r) were used to indicate the magnitude and direction of each linear relationship. The slopes of the regression lines in elderly and young groups were compared using analysis of covariance (ANCOVA). Differences between pre- and post-test values in FO_2_ and FCO_2_ during the recovery period were analyzed using one factor ANOVA with repeated measures. When significance was found, Student's *t*-test with Bonferroni correction for multiple comparisons was used to locate the significance. Significance was set at *P* < 0.05. Statistical analyses were performed using SPSS 17.0 (SPSS Inc., Chicago, USA). Data in the text, tables and figures are reported as mean ± SD.

## Results

### Exercise trials

As designed, the duration of the young cycling exercise trials (26:53 ± 3 min) was higher (*P* < 0.001) than the duration of the elderly treadmill exercise trials (9:29 ± 3 min). Maximal power output reached by young athletes was 294 ± 34 W (3.74 ± 0.54 W· kg^−1^). Maximal grade attained by elderly individuals at 6.1 km· h^−1^ was 8.5 ± 3.9%. Young athletes attained significantly higher (*P* < 0.001) peak heart rate and peak blood lactate concentration values (195 ± 11 b· min^−1^ and 10.3 ± 2.2 mmol·L^−1^) compared to elderly individuals (144 ± 24 b· min^−1^ and 6.6 ± 1.7 mmol·L^−1^, respectively).

The pre-test calibration and post-test verification values of FO_2_ and FCO_2_ of the room air and tank gases assessed within 15 s of the completion of each exercise trial in the whole group of subjects are presented in Table 2. The system showed a downscale drift (*P* < 0.001) in FO_2_ and FCO_2_ from pre- to post-test values in the exercise trials. Mean absolute differences between pre- and post-test values were −0.18% (room air) and −0.14% (tank gas) in O_2_ and 0.00% (room air) and −0.05% (tank gas) in CO_2_. Expressed as a percentage of the average pre-test calibration values, the magnitude of the downscale drift was similar (~0.9%) in both analyzers. There was no statistical difference (*P* = 0.08; 95% CI: −0.00 to 0.01 L) in the registered air volumes between post-test verification (2.99 ± 0.01 L) and pre-test calibration values (2.99 ± 0.01 L). This means that the calibration factor for ventilation volume was essentially constant throughout the test period.

**Table 2:** **Calibration (pre-test) and verification (post-test) readings of the exercise and non-exercise trials**.

	**Fractional oxygen concentration (%)**				**Fractional carbon dioxide concentration (%)**			
	**Room air**		**Tank gas**		**Room air**		**Tank gas**	
	**Pre**	**Post**	**Pre**	**Post**	**Pre**	**Post**	**Pre**	**Post**
**EXERCISE TRIALS, *N* = 30**								
Mean	20.93	20.75^**^	15.05	14.91^**^	0.00	0.00^**^	5.99	5.94^**^
SD	N/A	0.06	N/A	0.07	N/A	0.01	N/A	0.02
**RANGE**								
Min	N/A	20.61	N/A	14.82	N/A	0.00	N/A	5.89
Max	N/A	20.91	N/A	15.05	N/A	0.02	N/A	5.97
**NON-EXERCISE TRIALS, *N* = 30**								
Mean	20.93	20.93^††^	15.05	15.05^††^	0.00	0.01^*^^†^	5.99	6.00^*^^††^
SD	N/A	0.01	N/A	0.01	N/A	0.01	N/A	0.01
**RANGE**								
Min	N/A	20.90	N/A	15.03	N/A	0.00	N/A	5.99
Max	N/A	20.95	N/A	15.07	N/A	0.02	N/A	6.02

*Pre, pre-test calibration readings; Post, post-test verification readings*.

Significantly different from Pre:

* *P < 0.01*,

** *P < 0.001*.

Significantly different from the exercise trials:

† *P < 0.01*,

†† *P < 0.001*.

**Figure 2:**
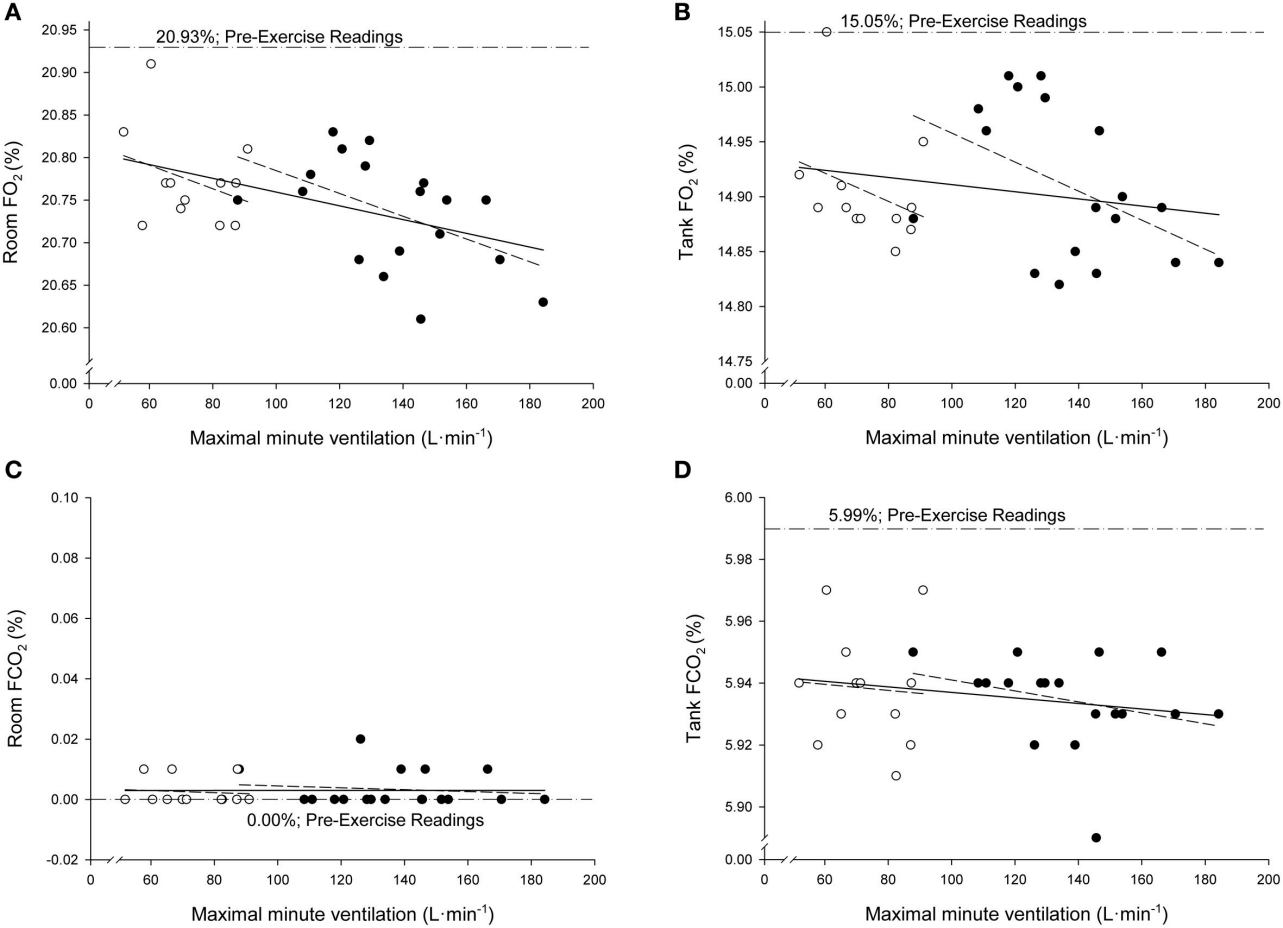
**Relationships between the individual values of maximal minute ventilation and the individual post-test verification values of fractional concentrations of oxygen (FO_2_; A,B) and carbon dioxide (FCO_2_; C,D) when both calibration gases (room air and tank gas) were run through the metabolic system after maximal exercise**. Open circles: elderly sedentary subjects. Filled circles: young athletes.

Figure 2 presents the relationships in the total sample between the individual values of ${\dot{\text{V}}}_{{\text{E}}_{\text{max}}}$ and the individual post-test verification values of FO_2_ and FCO_2_ of both calibration gases (room air and tank gas). Regression analyses indicated significant negative correlations between ${\dot{\text{V}}}_{{\text{E}}_{\text{max}}}$ and post-test verification values of room air FO_2_ in the total sample (*r* = −0.48; *P* = 0.007; SEE = 0.056%; 95% CI: 20.77–20.91%) and in the young group (*r* = −0.49; *P* = 0.03; SEE = 0.057%; 95% CI: 20.75–21.04%). The gradients of the rest of the relationships presented in Figure 2 were not different from zero (*P* < 0.05). According to the ANCOVA results, the slopes of the regression lines were not different among groups (*P* > 0.05). No significant relationships were observed between test duration and post-test verification values of FO_2_ and FCO_2_ (*P* > 0.05). No other relevant significance was found between respiratory parameters and post-trial readings.

### Non-exercise trials

During the non-exercise trials, the drift over time in the electronic gas analysis system was minimal because FO_2_ and FCO_2_ remained very stable throughout the time (Table 2). The highest individual difference in the post-test verification during the non-exercise trials was only of 0.03% in FO_2_ and of 0.02% in FCO_2_.

### Measured and corrected respiratory values

Measured F_E_O_2_ values reached at $\dot{\text{V}}{\text{o}}_{2\text{max}}$ during exercise by the young and elderly groups were 17.39 ± 0.29% and 17.18 ± 0.52%, respectively. When these values were corrected with the proposed correction equation, the corresponding F_E_O_2_ values at $\dot{\text{V}}{\text{o}}_{2\text{max}}$ were 17.54 ± 0.32% and 17.32 ± 0.53% for the young and elderly groups, respectively. Measured F_E_CO_2_ values reached at $\dot{\text{V}}{\text{o}}_{2\text{max}}$ by the young and elderly groups were 3.77 ± 0.28% and 4.00 ± 0.51%, respectively. When these values were corrected, the corresponding F_E_CO_2_ values at $\dot{\text{V}}{\text{o}}_{2\text{max}}$ were 3.80 ± 0.29% and 4.03 ± 0.50% for the young and elderly groups, respectively. Inasmuch as no drift was observed in the calibration factor for ventilation volume during exercise, there were no differences between corrected and measured values of ${\dot{\text{V}}}_{{\text{E}}_{\text{max}}}$ in any of the groups (*P* > 0.05). Average ${\dot{\text{V}}}_{\text{Emax}}$ was 88% higher (*P* < 0.001; 95% CI 49 to 80 L·min^−1^) in the young group compared with the elderly group (137 vs. 73 L·min^−1^).

**Figure 3:**
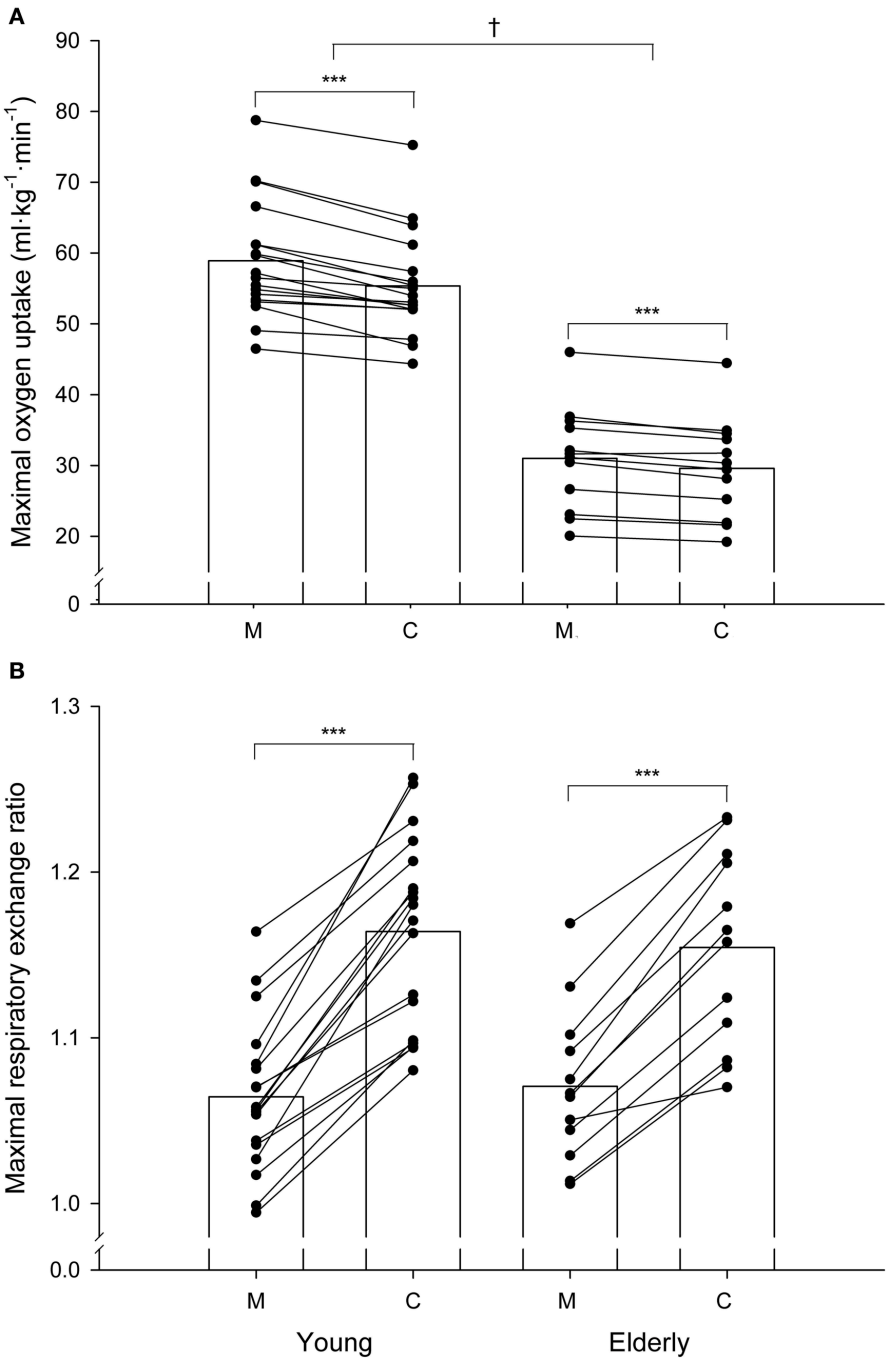
**Individual measured (M) and corrected (C) values of maximal oxygen uptake (A) and maximal respiratory exchange ratio (B) in the young and elderly groups**. Maximal oxygen uptake is expressed in ml·kg·min^−1^. The *bars* indicate mean values. ^***^Significant difference between the corrected and the corresponding measured values (*P* < 0.001). †Significant difference between groups (*P* < 0.05).

The corrected F_E_O_2_ and F_E_CO_2_ values resulted in systematic significant changes in $\dot{\text{V}}{\text{co}}_{2\text{max}}$ and $\dot{\text{V}}{\text{o}}_{2\text{max}}$ values. Measured $\dot{\text{V}}{\text{co}}_{2\text{max}}$ values reached by the young and elderly groups were 4.93 ± 0.57 L·min^−1^ and 2.82 ± 0.61% L·min^−1^, respectively. When these values were corrected, the average $\dot{\text{V}}{\text{co}}_{2\text{max}}$ values (5.07 ± 0.59 L·min^−1^ and 2.98 ± 0.62 L·min^−1^ for the young and elderly groups respectively) were 3–5% higher (*P* < 0.001) than the corresponding measured values. The measured average $\dot{\text{V}}{\text{o}}_{2\text{max}}$ values in the young and elderly groups were 4.64 ± 0.56 L·min^−1^ and 2.62 ± 0.50 L·min^−1^, respectively. Corrected average $\dot{\text{V}}{\text{o}}_{2\text{max}}$ values (4.35 ± 0.46 and 2.50 ± 0.47 L·min^−1^ for the young and elderly groups, respectively) were 5–6% lower (*P* < 0.001) than the corresponding measured values. The individual overestimation of the measured $\dot{\text{V}}{\text{o}}_{2\text{max}}$ values ranged from 0.3 to 11%. Figure 3A shows the average and the individual measured and corrected $\dot{\text{V}}{\text{o}}_{2\text{max}}$ values, expressed relative to kilogram of body mass, in the young and elderly subjects. Average corrected $\dot{\text{V}}{\text{o}}_{2\text{max}}$ values were 3.6 ml·kg^−1^·min^−1^ (young) and 1.4 ml·kg^−1^·min^−1^ (elderly) lower (*P* < 0.001) than the average measured $\dot{\text{V}}{\text{o}}_{2\text{max}}$ values. In every subject, the corrected $\dot{\text{V}}{\text{o}}_{2\text{max}}$ value was lower than the measured value.

Figure 3B shows the average and the individual measured and corrected RER_max_ values in the young and elderly subjects. The average measured RER_max_ values were 1.06 ± 0.05 in the young group and 1.07 ± 0.05 in the elderly group. When these values were corrected, the average RER_max_ values (1.16 ± 0.06 and 1.15 ± 0.06 for the young and elderly groups, respectively) were 8–9% higher (*P* < 0.001) than the corresponding measured values. In every subject, the corrected RER_max_ value was higher than the measured value.

When the measured values were taken into account, 14 out of the 18 young subjects (78%) and 9 out of the 12 old subjects (75%) satisfied at least two of the criteria established to verify attainment of $\dot{\text{V}}{\text{o}}_{2\text{max}}$. When the RER_max_ and the $\dot{\text{V}}{\text{o}}_{2\text{max}}$ values were corrected, the ratio of the subjects who met these criteria increased to 89 and 83% in the young and elderly groups respectively.

### Recovery trials

**Figure 4:**
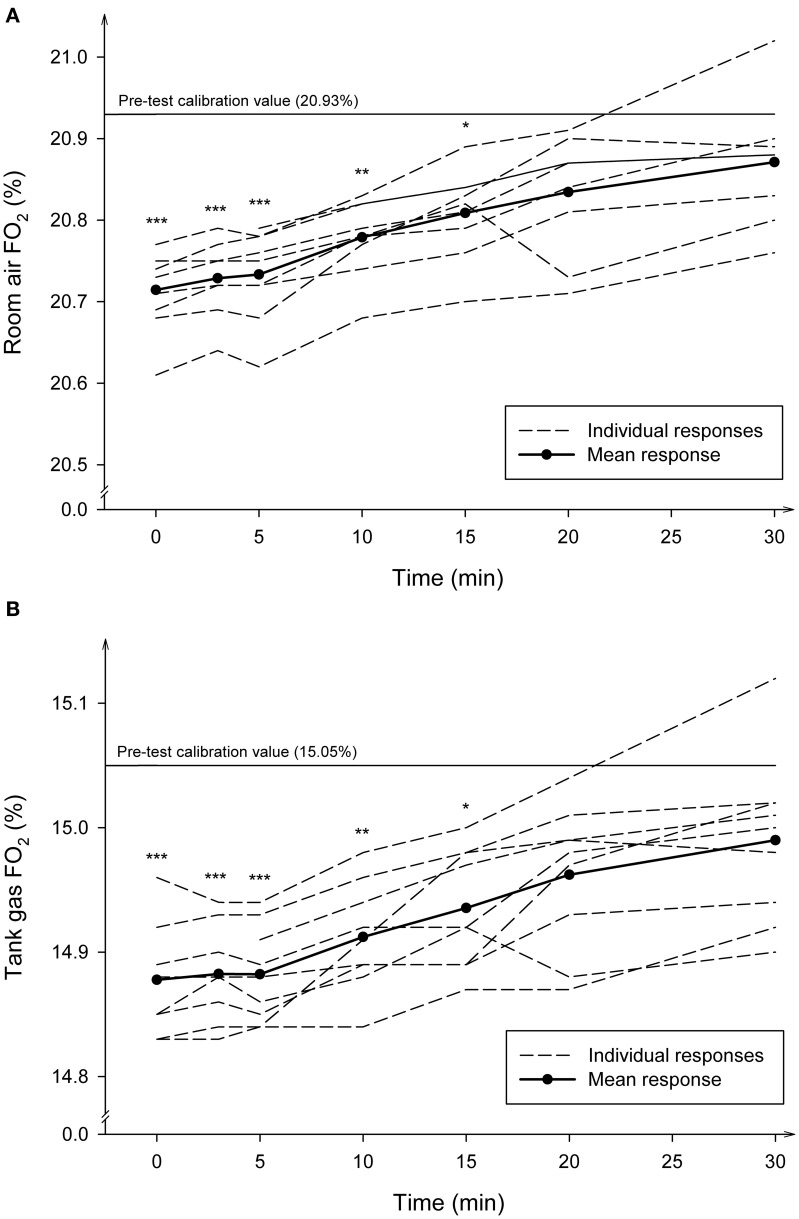
**Average and individual time course of the fractional concentrations of oxygen (FO_2_) during recovery after maximal exercise**. The post-test verification values read by the gas analyzers using the room air **(A)** and the tank gas **(B)** were measured at 20 s, 3, 5, 10, 15, 20, and 30 min of recovery. The number of observations made at each time-point was 9. ^***^Significantly different from pre-test (*P* < 0.001); ^**^Significantly different from pre-test (*P* < 0.01); ^*^Significantly different form pre-test (*P* < 0.05).

Figure 4 shows the average and individual FO_2_ changes observed in 9 subjects when the post-test verification process was repeated several times during the first 30 min of recovery after the completion of the exercise trials, and after disconnecting the gas sample line from the subjects' mask. During the first 5 min of recovery the average FO_2_ remained similar to the significantly diminished values (*P* < 0.001) read immediately after the end of the exercise trials. From that time on, the FO_2_ reading values increased progressively and linearly over the time. The disappearance of the drift was completed after 20 min of recovery, although at this time the average FO_2_ readings still tended to be slightly lower than the pre-test calibration values (*P* = 0.20). Similar patterns were observed for the time course of FCO_2_ changes (data not shown).

## Discussion

The main finding of this study is that the pre-test calibration and the post-test verification values of O_2_ and CO_2_ demonstrated a downscale drift in the O_2_ and CO_2_ readings. The drift was observed in all the exercise tests and was higher than the absolute accuracy of at least ±0.03% (Gore et al., 2013) and ±0.05% (Jones, 1988) that laboratories should strive to attain for electronic O_2_ and CO_2_ analyzers. This indicates that the present metabolic system systematically underestimates F_E_O_2_ and F_E_CO_2_ values during maximal exercise.

Several potential sources of error, working separately or together, could explain the F_E_O_2_ and F_E_CO_2_ downscale drifts during maximal exercise (Robergs et al., 2010). One potential source of error may be due to an electrical instability in the analyzers over time (Kannagi et al., 1983). Evidence of this mechanism, however, has not been provided. When a series of calibrations were assessed during the non-exercise pair-matched trials without any subject being connected to the metabolic system, the O_2_ and CO_2_ readings remained unchanged over the course of the period (Table 2). This suggests that no base-line drift of the analyzers occurred due to an electronic error, indicating that the analyzers were electrically stable for a long period of time.

The most likely factor explaining the reduction in O_2_ and CO_2_ percentages may be associated with how water vapor is handled in the aspirated gas by the analyser. The metabolic system used in the present study sends the exhaled gas to the O_2_ and CO_2_ gas analyzers through a built-in Nafion gas dryer humidifier conducting tube. This tube provides a constant value of water vapor tension of the exhaled and the calibration gases just prior to the entry of the samples into the gas analyzers. It is possible that the observed downscale drift could be partly explained by an incomplete removal of the water vapor tension of the aspirated gas by the analyser to equilibrate the partial water vapor pressure (PH_2_O) into and out of the Nafion tube wall. Since the O_2_ and CO_2_ analyzers are partial pressure sensors that measure gas fractions of the total gas volume including water vapor, and they are sensitive to the presence of water vapor molecules, the passage of excessive water vapor to the gas analyzers could raise the PH_2_O of the sample. A rise in PH_2_O would reduce O_2_ and CO_2_ fractions by the factor [(P_B_ − PH_2_O excess)·(P_B_)^−1^] or [(1 − FH_2_0)] (Gore et al., 2013) and the analyzer would read lower concentration values (Auchincloss et al., 1970). The observation that the O_2_ and CO_2_ drifts were almost completely reversed in a few min after exercise by simply disconnecting the sampling line from the flow-meter and the subject's mask, and by flushing the system with room air (Figure 4), supports the notion that some failure in the drying process occurred during exercise.

Under the assumption of an incomplete removal of the water vapor, it is possible to estimate the average extra amount of PH_2_O at a given temperature that was not removed by the Nafion tube to equilibrate the aspirated gas by the analyzers to the level of ambient humidity during exercise. This can be calculated from the average drift values observed in the O_2_ (from 20.93 to 20.75% and from 15.05 to 14.91%) and CO_2_ analyzers (from 5.99 to 5.94%) (Table 2) using the following formula (Bageant, 1976; Gore et al., 2013):

$$\begin{array}{l} \text{Read }{\text{O}}_{2}\%=\left[\text{True }{\text{O}}_{2}\%\cdot \left({\text{P}}_{\text{B}}-{\text{PH}}_{2}\text{O}\right)\right]\cdot {\left({\text{P}}_{\text{B}}\right)}^{-1} \end{array}$$

where read O_2_% is the oxygen percentage read during the post-test verification, true O_2_% is the oxygen percentage read during the pre-test calibration, and P_B_ is ambient barometric pressure (in our case: ~724 mmHg).

In that case, the estimated average PH_2_O that could not be removed was 6.2 mmHg (range 0.7–11 mmHg) for the O_2_ calibration with room air, 6.7 mmHg (range: 0–11 mmHg) for the O_2_ calibration with the tank, and 6.0 mmHg (range: 2.4–12.1 mmHg) for the CO_2_ calibration with the tank. Inasmuch as the PH_2_O of the exhaled gas leaving the body is ~47 mmHg (on the basis of ~100% of relative humidity, at body temperature) (Bageant, 1976), an incomplete average removal of around 6.3 mmHg of water vapor corresponds to ~13% of excess in relative humidity (6.3 · 100 · 47^−1^) that cannot be cleared from the circuit, with individual values ranging from 2 to 24%.

The reason why the Nafion tube could not fully equilibrate the gas being conveyed to the analyzers with the ambient humidity is unknown. However it can be related to:

1. A saturation process that reduces active surface area in the Nafion tubing. It is known that some saturation process occurs in the Nafion tubing since the wall of the tubing always retains some residual water, because the sulphonic acid groups within the Nafion polymer will never give up all their water (Mauritz and Moore, 2004). When the dryer becomes progressively physically wet over time, a failure to dry occurs. This failure to dry may be more relevant when the exhaled air flow is high and, therefore, when the aspirated gas sample's flow rate (0.5% of the exhaled flow rate) and its water vapor content are high. For example, in the young exercise trials the amount of water vapor content to be removed out of the Nafion tube can be 16 times higher at maximal exercise (exhaled flow gas: 190 L·min^−1^; aspired gas: 950 ml·min^−1^) than at rest (exhaled flow gas: 12 L·min^−1^; aspired gas: 60 ml·min^−1^). This is in agreement with the significant linear negative correlation observed in this study between ${\dot{\text{V}}}_{{\text{E}}_{\text{max}}}$ and the magnitude of the drift in FO_2_ (Figure 2). This strongly suggests that the higher the ${\dot{\text{V}}}_{\text{E}}$ and the amount of water vapor to be removed, the higher the absolute magnitude of the drift.
2. The inability of the system to maintain a very low water pressure outside, in the air surrounding the Nafion tube wall. An excess of condensate water vapor may be surrounding the Nafion tube as a consequence of the release of the excess of moisture out of the tube. This process may be more pronounced when the Nafion tube is located inside the metabolic measurement cart, such as in the metabolic system used in this study. In such a case, the fan of the metabolic cart cannot remove this excess water vapor condensed inside the metabolic cart.
3. Factors like the accumulation of sweat, saliva, foreign bodies and condensation generated by the subject can enter the internal lumen of the sampling line; a portion of exhaled air is drawn and, therefore, a change in the resistance of the delivery tubing or in the gas sampling rate can occur. This could contribute to a decrease in the gas flow rate and pressure in the sampling tube, leading to irregular results (Atkinson et al., 2005; Gore et al., 2013).

The present results support the above theoretical possibilities that cause an incomplete removal of water vapor of the aspired gas transported from the mouth to the analyzers before the gas enters the analyzers. This would explain the significant downscale drift in the O_2_ and CO_2_ analyzers that occurs during continuous measurement during human exercise.

The practical question to consider is the influence of the analyzers' drifts on $\dot{\text{V}}{\text{o}}_{2\text{max}}$. The correction used for the difference between the pre- and post-test conditions indicated that the corrected $\dot{\text{V}}{\text{o}}_{2\text{max}}$ values were on average 3.6 ml·kg^−1^·min^−1^ (young subjects) and 1.4 ml·kg^−1^·min^−1^ (older subjects) lower than those of the measured values. When expressed relative to the individual $\dot{\text{V}}{\text{o}}_{2\text{max}}$ values, the average difference between the measured and the corrected $\dot{\text{V}}{\text{o}}_{2\text{max}}$ values was similar (5–6%) in the young and the elderly subjects. This suggests that, in relative terms, there is a systematic and considerable overestimation in the measurement of $\dot{\text{V}}{\text{o}}_{2\text{max}}$ that is uniform over a full range of $\dot{\text{V}}{\text{o}}_{2\text{max}}$ values regardless of exercise duration. The average technological error of 5–6% may be considered unacceptable because it is larger than the ±0.5 to ±3% (technological error) or the ±2.2 to ±4% (technological plus biological variation) accuracy standards accepted for the precision of $\dot{\text{V}}{\text{o}}_{2\text{max}}$ measurement by most certifying organizations that supervise the accreditation process of the metabolic systems (American Thoracic Society, 1987; Gore et al., 2013). The present results may explain, at least partly, the reason why a measurement error of 5% in $\dot{\text{V}}{\text{o}}_{2\text{max}}$ between laboratories and metabolic systems is nowadays a difficult goal to achieve, owing to the combined technical error and the biological variation (Hodges et al., 2005).

A question raised is the comparison of the corrected $\dot{\text{V}}{\text{o}}_{2\text{max}}$ and RER_max_ data with published values. When compared to the measured values, the average corrected $\dot{\text{V}}{\text{o}}_{2\text{max}}$ values in the elderly group (29.6 ml·kg^−1^·min^−1^) and the average corrected $\dot{\text{V}}{\text{o}}_{2\text{max}}$-to cycling work rate values in the young group (14.7 ml O_2_·W^−1^) are lower than the measured values (31.0 ml·kg^−1^·min^−1^ and 15.7 ml O_2_·W^−1^), and compare favorably with those estimated for the elderly group (29.5 ml·kg^−1^·min^−1^) using the formula of the American College of Sports Medicine (2009) and with the average ratio (14.1 ml O_2_·W^−1^; range: 12.1–18.6) reported by other investigators using different metabolic systems during long duration (15–27 min) incremental maximal cycling tests (Pollock et al., 1982; Armstrong and Costill, 1985; Storer et al., 1990; American College of Sports Medicine, 2009; Bowen et al., 2012; Petot et al., 2012; Adami et al., 2013). The average corrected RER_max_ was 9% higher than the measured RER_max_ in the young group (1.16 vs. 1.06) and 8% higher in the elderly group (1.15 vs. 1.07). When RER_max_ values were not corrected, only 17% of the young and 25% of the elderly subjects reached a RER_max_ greater than 1.10, the most widely used secondary criterion to verify attainment of $\dot{\text{V}}{\text{o}}_{2\text{max}}$ (Howley et al., 1995; American College of Sports Medicine, 2009). A major effect of correcting the RER_max_ values was that the ratio of the subjects reaching a RER_max_ greater than 1.10 was increased to 72% in the young group and to 75% in the elderly group. The difference between the corrected and measured RER_max_ values suggests that some inconsistencies and failures found in several studies to satisfy RER_max_ criterion for achievement of $\dot{\text{V}}{\text{o}}_{2\text{max}}$ may be largely due to an artifact related to technological error (Bowen et al., 2012). This indicates that correction of $\dot{\text{V}}{\text{o}}_{2\text{max}}$ and RER_max_ values, on the basis of the F_E_O_2_ and F_E_CO_2_ drifts observed, produced more reasonable and satisfactory values than the measured ones.

This study has several limitations. The major drawback comes from the fact that we did not corroborate the validity of the correction method suggested. There is also a lack of consensus on which method is the most appropriate to assess the reliability and validity of $\dot{\text{V}}{\text{o}}_{2}$ measures (Salier Eriksson et al., 2012). The conventional Douglas bag procedure has been regarded as the gold standard method to validate metabolic measurement systems (McLaughlin et al., 2001; Rietjens et al., 2001). This method remains, however, very limited (Salier Eriksson et al., 2012). In any case, in close agreement with our corrected values, Medbø et al. (2002) and Larsson et al. (2004) found that a commercial metabolic system (Metamax II), utilizing a built-in Nafion conducting tube, significantly overestimated $\dot{\text{V}}{\text{o}}_{2}$ by 4–13% and underestimated RER by 6% compared to the Douglas bag method. However, other validation studies have produced more varied results (Versteeg and Kippersluis, 1989; Bassett et al., 2001; McLaughlin et al., 2001). An alternative method to validate $\dot{\text{V}}{\text{o}}_{2}$ and RER measures is to use a metabolic calibrator system. However, the external validity of such a test is limited since it often uses dry gases and does not involve challenging factors such as humidified gases and irregular breathing patterns (Macfarlane, 2001). In the absence of a reliable gold standard method, the rationale for the analyzer's drift correction method used in this study is that the time point at $\dot{\text{V}}{\text{o}}_{2\text{max}}$, which was reached close to the end of the test, is close to the time point at which the post-test verification was undertaken (within 15 sec of the end of each test). It seems, therefore, justifiable to remove and correct the variations observed in F_E_O_2_ and F_E_CO_2_ at $\dot{\text{V}}{\text{o}}_{2\text{max}}$ by adjusting the analyzer pre-exercise base-line values to the post-exercise verification values.

Another limitation of this study is that we used a single metabolic system. Therefore, the generalizability of our findings is constrained to the metabolic cart and the analyzers used. However, four studies reporting the average numerical downscale drifts in O_2_ immediately after exercise using other metabolic systems have found values ranging from −0.02 to −0.22% (Wilmore et al., 1976; Armstrong and Costill, 1985; Prieur et al., 1998; Rietjens et al., 2001). This indicates that an absolute downscale drift also occurs in other metabolic systems. If the main source of the error is related to the built-in Nafion gas dryer humidifier conducting tube, a lower error (or none) should occur when gas fractions are measured as fractions of dry gas, when ambient relative humidity is higher than in the present study (e.g., 60%) or when the condensate water vapor surrounding the Nafion tube is more efficiently removed. A wider study is needed to extend the present findings to the wide metabolic systems' population.

## Conclusion and perspectives

In conclusion, the present experiment indicates that, under controlled laboratory conditions, a physiologically significant downscale drift in FO_2_ and FCO_2_ was observed over time at the end of maximal exercise in elderly sedentary and young athletes using a metabolic cart equipped with a built-in Nafion conducting tube. The most likely explanation for the drift is an accumulation of excess water vapor in the sample line which could not be completely removed during transit through the Nafion conducting tube. The correction method proposed indicates that ignoring the effects of the drift would induce an average $\dot{\text{V}}{\text{o}}_{2\text{max}}$ overestimation of 5–6% and a RER_max_ underestimation of 8–9%, with errors ranging up to 11–12% ($\dot{\text{V}}{\text{o}}_{2\text{max}}$) and up to 15–16% (RER_max_). Therefore, ignoring the drift can have an important influence on the accurate calculation of these variables. The disagreement between the measured and the corrected $\dot{\text{V}}{\text{o}}_{2\text{max}}$ and RER_max_ values observed in this particular metabolic system is not acceptable to test athletes, to prescribe exercise intensities, to calculate the fat oxidation rate from RER values, or to use the respiratory values for some other clinical purposes, such as to guide treatment in patients with chronic heart failure (Bowen et al., 2012), to enter in cardiac transplantation listing, to indicate the health status or to predict prognosis and mortality (Myers et al., 2002; Mehra et al., 2006). The implications of the present study point to the necessity to check FO_2_ and FCO_2_ values by carefully calibrating the pre-test calibration gases and verifying a possible shift immediately after exercise, as well as to correct the respiratory data in situations where the drift in O_2_ and CO_2_ analyzers occurs. Special care must be taken in studies where a Nafion conducting tube is used. Further research in this area is certainly warranted to establish valid correction factors for each device.

## Author contributions

EG, IG, and JE conceived and designed the experiments; EG, IG, and JA contributed to the acquisition and analysis of the data; EG, IG, JE, and JA interpreted the data; EG wrote the first draft; EG, IG, JE, and JA critically reviewed and edited the drafts; all authors approved the final version of the manuscript.

### Conflict of interest statement

The authors declare that the research was conducted in the absence of any commercial or financial relationships that could be construed as a potential conflict of interest.
